# On the existence conditions of interaction indices in response surface models

**DOI:** 10.1007/s10928-026-10026-x

**Published:** 2026-03-02

**Authors:** Erhan Yumuk, Clara Ionescu

**Affiliations:** 1 Department of Electromechanics, Systems and Metal Engineering, Research lab on Dynamical Systems and Control, Tech Lane Science Park 125, Ghent University, Ghent, 9052 Belgium; 2 Department of Control and Automation Engineering, Faculty Electrical, Electronics Faculty, Istanbul Technical University, Istanbul, Turkey; 3 Core lab MIRO, Flanders Make@UGent, Ghent, Belgium

**Keywords:** Response surface models, Interaction index, Feasibility analysis, Singular sets, Nonlinear systems

## Abstract

Response-surface models (RSMs) are widely used to characterize the combined effects of two agents and to classify their interaction as additivity, synergy, or antagonism. In this context, an interaction index is frequently used as a quantitative measure. However, estimation of the interaction index outside feasible ranges may result in complex-valued or biologically implausible predictions in certain regions of the input domain, thereby limiting the validity of the model. This paper introduces a feasibility analysis framework that distinguishes between isobole-level feasibility (i.e. the existence of well-defined isoboles at a given effect level) and global feasibility (i.e. well-posedness across the entire domain). The analysis explicitly characterizes the singular sets that arise when feasibility conditions are violated, thereby explaining when and how models fail. The approach is demonstrated on three canonical models, i.e. Greco, Minto, and Finney, and supported by numerical illustrations, offering practical guidance for systematic and robust parameter selection in drug combination studies, toxicology, and process engineering.

## Introduction

Response surface models (RSMs) provide a versatile framework for analyzing the combined effects of multiple agents across diverse domains, including pharmacology, toxicology, environmental sciences, and process engineering [1–3]. By linking agent doses to observed responses, such models enable systematic assessment of additivity, synergy, and antagonism. Foundational concepts such as Loewe additivity [4] and Bliss independence [5] laid the theoretical groundwork for combination assessment, while more advanced formulations including the Greco [6], Minto [7], Plummer [8], and Finney models [9], extend these ideas by introducing explicit interaction indices and flexible isobole geometries. These advanced formulations have become standard for interpreting experimental data and guiding rational design in combination studies.

The Hill equation, as one of the earliest and most influential models in quantitative pharmacology, underpins many of these frameworks and has been extensively studied both from a theoretical and a systems biology perspective [10–13]. Beyond its classical use, it has also inspired modern interaction models and extensions into systems biology and nonlinear dynamics [14]. Moreover, its role as a building block in more complex response surface formulations has provided a bridge between fundamental biochemical principles and practical modeling of drug interactions. In particular, RSMs have become standard tools in anesthesia research [15, 16] and are increasingly used to analyze multi-drug combinations in preclinical and clinical studies [17, 18]. More recent developments have further expanded their scope into computational pharmacology, including drug–drug and drug–target interaction prediction [19, 20], as well as physiologically based pharmacokinetic modeling [21], highlighting the growing relevance of these models for data integration and predictive medicine. Beyond biomedicine, RSM has also found widespread applications in process engineering, including optimization of ash digestion processes [22] and biosorption studies [23], underscoring its versatility as a general multivariable modeling framework.

Despite its broad usability, several limitations of traditional formulations have been recognized. In particular, log-agent effect models introduced by Plummer [8] and Finney [9], and later extended by Kong [24], rely on the assumption of constant relative potency, which is often overly restrictive. Furthermore, representing agent interaction with a single parameter can be inadequate when synergy, additivity, and antagonism coexist in different regions of the dose–response surface. These challenges have motivated the development of more flexible approaches, such as dose-dependent interaction functions that can better capture heterogeneous behaviors across the drug combination space. Similar concerns have also been raised in applied domains such as pharmaceutical development, where response surface methodology has been employed as a practical optimization tool [25].

Despite their widespread adoption, RSMs can exhibit mathematical infeasibility in certain parameter regimes. In particular, inappropriate values of the interaction index may yield unfeasible regions or singular sets, limiting the interpretability and predictive value of the model. Although such phenomena have been noted in practice, a systematic framework for characterizing feasibility across different model classes has been lacking. As these models are increasingly applied across domains including drug development, toxicology, and process engineering, a deeper understanding of their feasibility properties has become essential for reliable application.

The present work addresses this gap by developing a feasibility analysis framework for response-surface interaction models. We distinguish between isobole-level feasibility (existence of valid isoboles at a given effect level) and global feasibility (well-posedness over the entire input domain). For each of the three models, i.e. Greco, Minto, and Finney, we characterize the conditions under which feasible solutions exist and explicitly identify the singular sets that arise when feasibility is violated. Numerical illustrations confirm the theoretical results and provide practical guidance for robust parameter selection and model interpretation.

The remainder of this paper is organized as follows. Section “Methods” reviews the response surface models considered in this study. Moreover, presents the feasibility analysis, including theorems and corollaries for each model. Section “Results” provides numerical examples illustrating feasible, boundary, and infeasible scenarios. Section “Discussion” discusses the main findings of the paper and outlines directions for future work.

## Methods

### Response surface models

#### Hill and median-effect functions

The Hill function (also known as the *Emax model*) is widely used to describe single-agent input–response relationships. It is defined as:

1 $$\begin{aligned} E = E_{max} \frac{\left( \frac{c}{C_{50}}\right) ^\gamma }{1+\left( \frac{c}{C_{50}}\right) ^\gamma } , \end{aligned}$$

where *c* is the input level of agent, e.g. drug concentration, $C_{50}$ is the median effective level (the input producing 50% of the maximum effect), $E_{max}$ denotes the maximum achievable effect, and *γ * is the Hill coefficient that determines the slope of the curve.

A frequently used special case is obtained by normalizing the maximum effect, i.e. setting $E_{max}=1$. This yields the median-effect equation:

2 $$\begin{aligned} E = \frac{\left( \frac{c}{C_{50}}\right) ^\gamma }{1+\left( \frac{c}{C_{50}}\right) ^\gamma } \end{aligned}$$

The median-effect equation can be further transformed into a linear form using the median-effect linearization, i.e. taking *łog (E/(1-E))* against *łog (c)*:

3 $$\begin{aligned} Y=\log \left( \frac{E}{1-E}\right) =\gamma (\log (c) - \log (C_{50}))= \beta _0 + \beta _1\log (c) \end{aligned}$$

with $\beta _0 = -\gamma \log (C_{50})$ and $\beta _1= \gamma$. This linearization establishes a direct relationship between the transformed response *Y* and the logarithm of the input level *c*, which is convenient for both parameter estimation and model comparison.

This linearization allows to introduce the Loewe model to characterize the interaction between two agents:

4 $$\begin{aligned} \frac{c_1}{C_{y,1}} + \frac{c_2}{C_{y,2}} {\left\{ \begin{array}{ll} = 1 & \text {Additivity} \\ < 1 & \text {Synergy} \\ > 1 & \text {Antagonism} \end{array}\right. } \end{aligned}$$

where $c_1$ and $c_2$ are the input levels (e.g., concentrations or application intensities) of agent 1 and agent 2 that in combination produce the effect level *y*, while $C_{y,1}$ and $C_{y,2}$ are the corresponding input levels of each agent that yield the same effect *y* when applied individually. It is defined as:

5 $$\begin{aligned} C_{y,i} = C_{{50}_i} \left( \frac{y}{1-y}\right) ^\frac{1}{\gamma _i}, \quad i =1,2 \end{aligned}$$

where $C_{{50}_i}$ denotes the median effective level for each agent, and $\gamma _i$ represents the Hill coefficient for each agent.

**Fig. 1:**
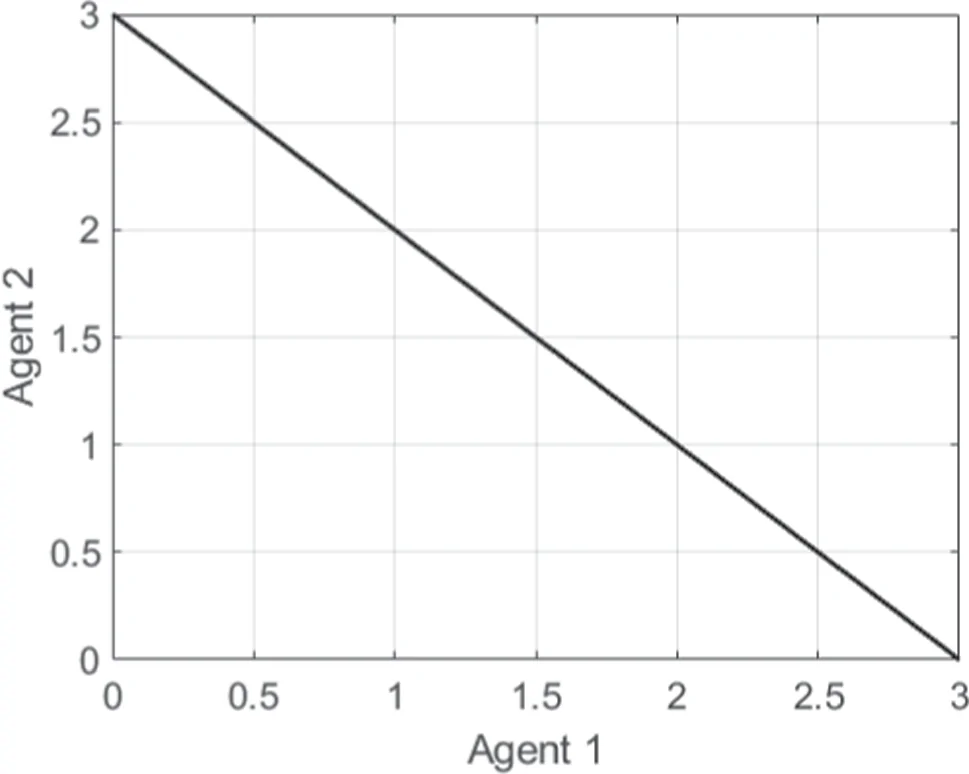
Isobologram of the Loewe additivity model (*y=0.5*), with parameters $C_{{50}_1}=C_{{50}_2}=3, \gamma _1 = \gamma _2 = 5$

The concept is commonly illustrated with an isobologram, where the additivity condition is represented by a straight line connecting the single-agent effect doses, as depicted in Fig. 1. Deviations below this line correspond to synergy, while deviations above it correspond to antagonism.

This representation provides the foundation for modeling multi-drug interactions under the principle of Loewe additivity, which in clinical medicine is applied to drugs within a therapy and is further extended through response surface models in the next subsection.

#### The Greco model

Assuming that the input–response curves of both agents follow the median-effect equation in Eq. 2, Greco et al. [6] proposed the following isobole equation corresponding to an effect *y*:

6 $$\begin{aligned} 1 = \frac{c_1}{C_{{50}_1}\left( \frac{y}{1-y}\right) ^\frac{1}{\gamma _1}}+\frac{c_2}{C_{{50}_2}\left( \frac{y}{1-y}\right) ^\frac{1}{\gamma _2}}+\frac{\beta c_1 c_2}{C_{{50}_1 C_{{50}_2}}\left( \frac{y}{1-y}\right) ^{\frac{1}{2 \gamma _1}+\frac{1}{2\gamma _2}}} \end{aligned}$$

where the parameter *β * quantifies the degree of interaction, i.e. synergism, additivity, or antagonism. When $\gamma _1=\gamma _2=\gamma$, this formula is reorganized and can be generalized as in [26]

7 $$\begin{aligned} E = \frac{ I^\gamma (c_1,c_2)}{1+ I^\gamma (c_1,c_2)} \end{aligned}$$

where the normalized index is

$$\begin{aligned} I(c_1,c_2)=\frac{c_1}{C_{{50}_1}} + \frac{c_2}{C_{{50}_2}} + \beta \frac{c_1}{C_{{50}_1}} \frac{c_2}{C_{{50}_2}}. \end{aligned}$$

**Fig. 2:**
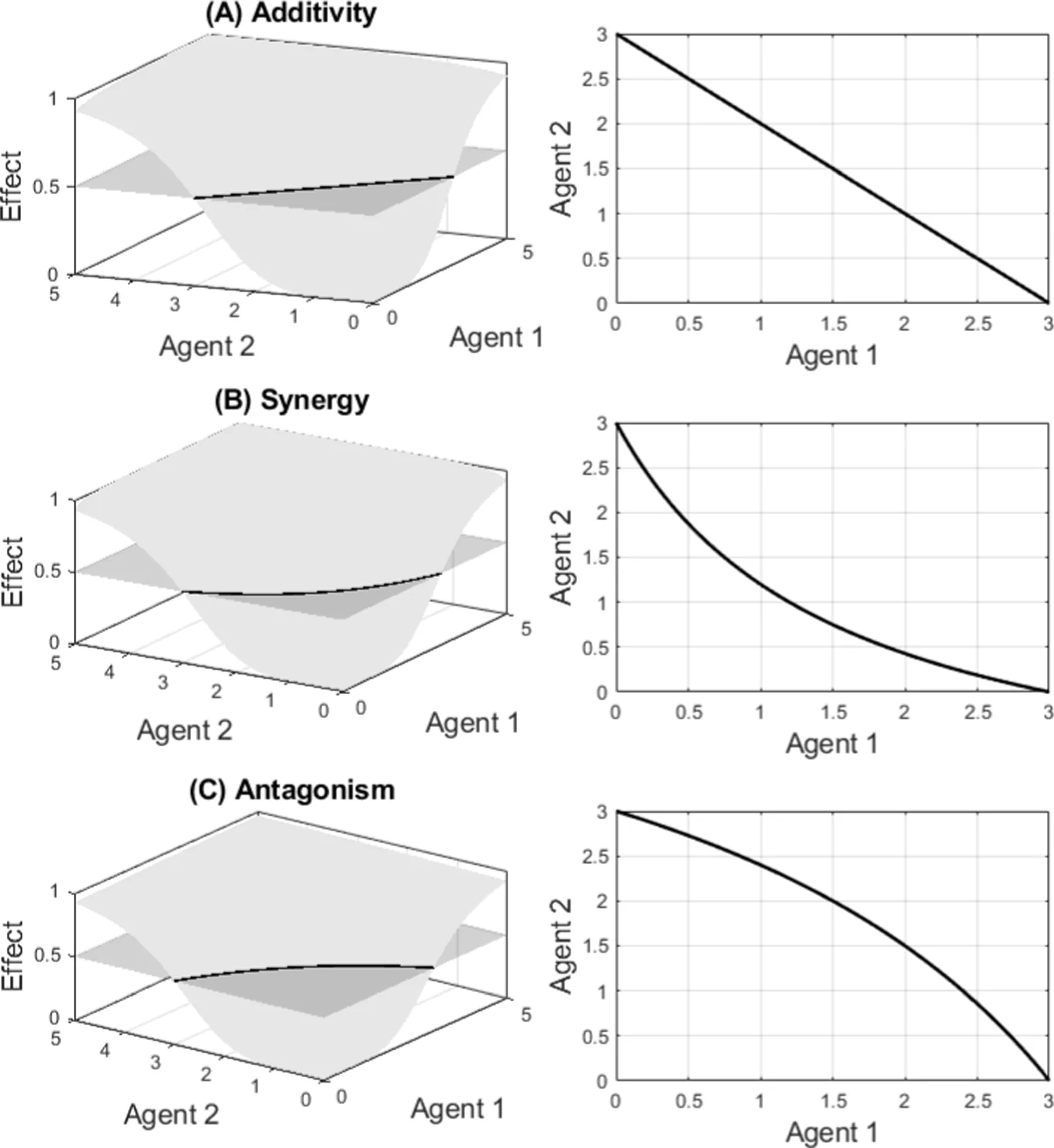
Greco model representation of additivity, synergy, and antagonism, with isoboles shown at effect level *y=0.5*. Parameters: $C_{{50}_1}=C_{{50}_2}=3, \gamma _1 = \gamma _2 = 5$

The interaction parameter is *β *, which captures the magnitude of synergy, additivity, or antagonism. For * β = 0*, the model represents *additivity*; for *β >0*, it indicates *synergistic* interaction; and for *β <0*, it corresponds to *antagonistic* interaction. The three situations along with their respective isoboles at effect level *y=0.5*, are illustrated in Fig. 2.

#### The Minto model

Minto et al. [7] introduced an alternative formulation by normalizing the input levels of each agent to their respective potencies. Their key idea was to treat any fixed ratio of the two agents as a “new entity,” which is characterized by its own sigmoidal $E_{max}$. Accordingly, a normalized index is defined as:

8 $$\begin{aligned} I(c_1,c_2)=\frac{I_1(c_1)+I_2(c_2)}{U_{50}(\theta )} \end{aligned}$$

where

9 $$\begin{aligned} U_{50}(\theta )= 1- \beta \theta +\beta \theta ^2, \quad I_1(c_1)=\frac{c_{1}}{C_{{50}_1}}, \quad I_2(c_2)=\frac{c_{2}}{C_{{50}_2}} \end{aligned}$$

Here, $$I_1(c_1)$$ and $$I_2 (c_2) $$ are the normalized ratios of input levels of agent, $$c_{1}$$ and agent 2, $$c_{2}$$ with respect to their median effective levels $$C_{{50}_1}$$ and $$C_{{50}_2}$$, respectively. The term *β * indicates the interaction as in the Greco model. In addition to the features of the Greco model, the potency of the drug mixture *θ * that ranges to 1 (only agent 1) from 0 (only agent 2) can be obtained as follows:

10 $$\begin{aligned} \theta (c_1, c_2) = \frac{I_1(c_1)}{I_1(c_1)+I_2(c_2)} \end{aligned}$$

#### The Finney model

The model proposed by Finney [9] is expressed as:

11 $$\begin{aligned} Y = \beta _0 + \beta _1 \log \left( c_1 + \rho c_2 + \beta _4 (\rho c_1 c_2)^\frac{1}{2}\right) , \end{aligned}$$

where *Y* denotes the transformed effect, $c_1$ and $c_2$ are the amounts of agents 1 and 2 in the combination, respectively, and $\beta _4$ represents the interaction term. This model assumes a fixed relative potency *ρ *, defined as $\rho = \frac{C_{{50}_1}}{C_{{50}_2}}$. Later, Plummer and Short [8] generalized this model to allow the relative potency *ρ * to vary, extending its applicability for more flexible interaction assessments.

### Analytical feasibility analysis

This section investigates the conditions under which response surface models yield real-valued and biologically interpretable responses under standard PKPD constraints. We begin by stating the basic assumptions on the input variables, model parameters, and transformations. These are followed by remarks that highlight immediate implications for the response and its transformed variables. Building on these observations, we establish a general feasibility condition as a proposition, which serves as the basis for the detailed analysis of each surface formulation in the subsequent sections.

#### Assumption 1

The input levels satisfy $c_1, c_2 > 0$, and the median effect doses are positive, i.e. $C_{{50}_1}, C_{{50}_2} > 0$.

#### Assumption 2

The Hill coefficients in Eq. 2 are strictly positive, i.e. $\gamma _1, \gamma _2, \gamma > 0$.

In the PKPD context, Assumptions 1 and 2 are naturally satisfied, since drug concentrations, their effects and Hill coefficient are intrinsically non-negative. Therefore, within PKPD applications these conditions should be interpreted as remarks rather than restrictive modeling assumptions. They are stated explicitly here to preserve generality of the response surface modeling framework and to accommodate potential applications in other domains where such positivity cannot be taken for granted.

Based on these assumptions, several immediate mathematical consequences can be derived regarding the model parameters and the domains of response and their transformations.

#### Remark 1

Based on Assumption 2, the slope parameter $\beta _1$ in Eq. 3 is strictly positive, i.e., $\beta _1 > 0$.

#### Remark 2

Based on Assumptions 1 and 2, the intercept parameter $\beta _0$ in Eq. 3 is strictly negative, i.e., $\beta _0 < 0$.

#### Remark 3

The response variable * E * in Eq. 2 is bounded within the interval *[0,1)*.

#### Remark 4

The transformed variable * Y * in Eq. 3 is well-defined only when * E ∈ (0,1) *.

These observations lead to a general feasibility condition, summarized in the following proposition.

#### Proposition 1

For all input pairs $(c_1,c_2)$ such that $I(c_1,c_2)$ is non-negative, the Hill surface model yields real-valued responses that are interpretable under domain-specific constraints; in PKPD applications, this corresponds to biological interpretability.

This condition forms the foundation for all global and local feasibility analyses of the specific surface formulations presented hereafter.

#### Proof

Recall that the Hill surface response is given in Eq. 7. For $I(c_1,c_2) \ge 0$ and *γ > 0*, the numerator is non-negative and the denominator strictly positive. Hence, the response *E* is real-valued and bounded in [0, 1), which corresponds to biological interpretability in PKPD applications. If $I(c_1,c_2)<0$, the exponentiation may become undefined or complex for non-integer *γ *, yielding biologically implausible values. Thus, the condition $I(c_1,c_2)\ge 0$ with *γ >0* guarantees real and interpretable responses, forming the basis for the feasibility analyses.*□ *

The subsequent subsections introduces a feasibility analysis that distinguishes between isobole-level feasibility (i.e. the existence of well-defined isoboles at a given effect level) and global feasibility (i.e. well-posedness across the entire domain).

#### Feasibility of the Greco model

##### Theorem 1

**(Global feasibility)** Consider the Greco model in Eq. 7. The model admits global feasibility for all $(c_1,c_2)>0$ if and only if $\beta \ge 0$.

##### Proof

We examine the sign of $I(c_1,c_2)$ to assess global feasibility.

- **Case A (**$\beta \ge 0$**).** For any $c_1,c_2>0$, the normalized index satisfies $I(c_1,c_2)>0$. Consequently, the effect level *E ∈ (0,1)* is well-defined for all $(c_1,c_2)>0$.
- **Case B (***β < 0***)** Along the diagonal $c_1=c_2\rightarrow \infty$, the normalized index becomes
  $$ I(c_1,c_1) = 2c_1 + \beta c_1^2 \;\rightarrow \; -\infty . $$
  Hence, *I* attains negative values. In this case, $I^\gamma <0$ or a complex value if *γ * is different from even integer. In this case, *E* is no longer biologically interpretable. Therefore, no global feasibility holds when *β <0*.

It follows that the Greco model is globally feasible for all $(c_1,c_2)>0$ if and only if $\beta \ge 0$.*□ *

##### Theorem 2

**(Isobole-level feasibility)** The Greco model produces real, feasible values provided that the interaction parameter *β * satisfies

$$ \beta> -\frac{C_{{50}_1}}{c_{y_1}} \quad \text {or} \quad \beta > -\frac{C_{{50}_2}}{c_{y_2}} . $$

##### Proof

Consider the isobole corresponding to a fixed effect level *y*. The corresponding normalized index is

$$ I_y = \Big (\tfrac{y}{1-y}\Big )^{\tfrac{1}{\gamma }} $$

- **Case A (fixing** $c_1$**)**. Solving for $c_2$ gives
  $$ c_2 = \frac{ I_y - \tfrac{c_1}{C_{{50}_1}}}{\tfrac{1}{C_{{50}_2}} + \beta \tfrac{c_1}{C_{{50}_1}} \tfrac{1}{C_{{50}_2}}} . $$
  The denominator vanishes when
  $$ \frac{1}{C_{{50}_2}} + \beta \frac{c_1}{C_{{50}_1}} \frac{1}{C_{{50}_2}} = 0, $$
  which yields $\beta = -C_{{50}_1}/c_{1}$. Since $c_1 \in [0, c_{y_1}]$, $\beta \in [-\frac{C_{{50}_1}}{c_{y_1}}, -\infty ]$. It follows the feasible condition:
  $$ \beta > -\frac{C_{{50}_1}}{c_{y_1}} . $$
- **Case B (fixing** $c_2$**).** Analogously, solving for $c_1$ gives
  $$ c_1 = \frac{I_y - \tfrac{c_2}{C_{{50}_2}}}{\tfrac{1}{C_{{50}_1}} + \beta \tfrac{c_2}{C_{{50}_2}} \tfrac{1}{C_{{50}_1}}} . $$
  The denominator vanishes when
  $$ \frac{1}{C_{m_1}} + \beta \frac{c_2}{C_{{50}_2}} \frac{1}{C_{{50}_1}} = 0, $$
  which yields $\beta = -C_{{50}_2}/c_{2}$. Since $c_2 \in [0, c_{y_2}]$, $\beta \in [-\frac{C_{{50}_2}}{c_{y_2}}, -\infty ]$. Then, the feasible condition is
  $$ \beta > -\frac{C_{{50}_2}}{c_{y_2}} . $$

When $\gamma _1 = \gamma _2$, we have $-C_{{50}_1}/c_{y1} = -C_{{50}_2}/c_{y2}$ using Eq. 5.*□ *

##### Corollary 1

**(Singularity lines of the Greco model)** The Greco model becomes singular along the vertical and horizontal lines

$$ c_1 = -\frac{C_{{50}_1}}{\beta }, \qquad c_2 = -\frac{C_{{50}_2}}{\beta }. $$

For *β >0*, these singularity lines lie in the negative region and thus outside the domain ($c_1,c_2>0$). For *β <0*, the lines intersect the positive region.

The above results clarify the feasibility domain of the Greco model. The global condition $\beta \ge 0$ ensures that all the combinations $(c_1,c_2)>0$ yield well-defined and biologically interpretable responses, corresponding to non-antagonistic interactions. When *β < 0*, feasibility is restricted to certain regions of the dose–effect surface, as captured by the isobole-level conditions. In this case, singularity lines appear within the positive quadrant, reflecting parameter choices for which the Greco formulation yields biologically implausible or undefined responses. These insights indicate that while the Greco model can represent synergistic and antagonistic interactions, its practical applicability in clinical or pharmacological settings requires respecting specific parameter constraints to ensure biological interpretability across the relevant domain.

#### Feasibility of the Minto model

##### Theorem 3

**(Global feasibility)** Consider the Minto model with $I_1(c_1) > 0$, $I_2(c_2) > 0$, and $\theta \in [0,1]$. Singularities occur if and only if $\beta \ge 4$. In that case, the singularity points exist at

$$ \theta _{\pm } = \frac{1 \pm \sqrt{1 - \tfrac{4}{\beta }}}{2}, $$

which both lie strictly within [0, 1].

##### Proof

The singularity condition is given by $U_{50}(\theta )=0$, i.e.,

$$ \beta \theta ^2 - \beta \theta + 1 = 0. $$

The discriminant is $\Delta = \beta ^2 - 4\beta = \beta (\beta -4)$.

- If *0< β < 4*, then *Δ < 0*, so no real roots exist.
- If *β = 4*, then *Δ = 0*, yielding one double root at $\theta =\tfrac{1}{2}$.
- If *β > 4*, then *Δ > 0*, producing two distinct real roots
  $$ \theta _{\pm } = \frac{1 \pm \sqrt{1 - \frac{4}{\beta }}}{2}, $$
  and both satisfy $\theta _{\pm } \in [0,1]$.
- If *β < 0*, then *Δ > 0* but the corresponding roots satisfy $\theta _{-}<0$ and $\theta _{+}>1$, hence they lie outside the the biologically interpretable domain.

Therefore, singularities arise if and only if $\beta \ge 4$.*□ *

##### Corollary 2

**(Singularity lines in the** $(I_1,I_2)$ **plane)** Under the assumptions of Theorem 3 with $\beta \ge 4$, the singular set in the $(I_1,I_2)$ plane consists of singular line(s) through the origin:

$$ I_2 = m_\pm \, I_1,\qquad m_\pm =\frac{1-\theta _\pm }{\theta _\pm } =\frac{1 \mp \sqrt{\,1-\tfrac{4}{\beta }\,}}{1 \pm \sqrt{\,1-\tfrac{4}{\beta }\,}}, $$

where $$\displaystyle \theta _\pm =\frac{1\pm \sqrt{1-\tfrac{4}{\beta }}}{2}$$.

##### Proof

Using Eq. 10,

$$ \theta =\frac{I_1}{I_1+I_2}=\theta _\pm \in (0,1). $$

Solving for $I_2$ gives

$$ I_2=\frac{1-\theta _\pm }{\theta _\pm }I_1. $$

Writing $\theta _{\pm } = \frac{1 \pm \sqrt{1 - \frac{4}{\beta }}}{2}$ yields $m_\pm =\frac{1\mp \sqrt{1 - \frac{4}{\beta }}}{1\pm \sqrt{1 - \frac{4}{\beta }}}$. For *β =4*, *m=1*. Then, this reduces to the single line $I_2=I_1$.*□ *

##### Corollary 3

**(Mapping of singularities to the** $(c_1,c_2)$ **plane)** For $I_1=\tfrac{c_1}{C_{{50}_1}}$ and $I_2=\tfrac{c_2}{C_{{50}_2}}$, the singular sets map to straight lines through the origin in the input level plane:

$$ c_2 \;=\; \kappa _\pm \, c_1,\qquad \kappa _\pm \;=\; \frac{C_{{50}_2}}{C_{{50}_1}}\, m_\pm \;=\; \frac{C_{{50}_2}}{C_{{50}_1}}\, \frac{1 \mp \sqrt{\,1-\tfrac{4}{\beta }\,}}{1 \pm \sqrt{\,1-\tfrac{4}{\beta }\,}} \, . $$

##### Proof

By substituting $I_1 = \tfrac{c_1}{C_{{50}_1}}$ and $I_2 = \tfrac{c_2}{C_{{50}_2}}$ into Corollary 2, the feasibility condition can be expressed in terms of the input variables $(c_1,c_2)$. For the specific case of *β =4*, this condition simplifies to the single line

$$ c_2 = \frac{C_{{50}_2}}{C_{{50}_1}}\,c_1, $$

which characterizes the corresponding singularity in the input space.*□ *

##### Theorem 4

**(Isobole-level feasibility)** For any fixed effect level *y ∈ (0,1)*, the corresponding isobole

$$ \frac{I_1+I_2}{U_{50}(\theta )} = I_y, \qquad I_y>0, $$

is well-defined if and only if $U_{50}(\theta ) > 0$ for all admissible $\theta \in (0,1)$.

Hence, isobole-level feasibility coincides with the global feasibility condition given in Theorem 3.

The feasibility analysis of the Minto model indicates that its validity depends critically on the value of the interaction parameter *β *. When *β < 4*, the model remains globally feasible with no singularities, ensuring well-defined drug–drug response surfaces. For $\beta \ge 4$, however, singularities arise along specific lines in both the normalized $(I_1,I_2)$ space and the input $(c_1,c_2)$ plane, leading to regions where the model loses biological interpretability. Hence, while the Minto model can capture a broad range of drug interactions, its reliable application requires carefully avoiding parameter regimes that induce singular behaviour.

#### Feasibility of the Finney model

##### Theorem 5

**(Global feasibility)** For all $c_1,c_2>0$ and *ρ >0*, the log-argument

$$ \Phi (c_1,c_2):=c_1+\rho c_2+\beta _4\sqrt{\rho c_1 c_2} $$

is strictly positive for every $(c_1,c_2)$ if and only if

$$ \quad \beta _4>-2. \quad $$

##### Proof

Let $x=\sqrt{c_1}$, $v=\sqrt{\rho c_2}>0$. Then $\Phi (x,v)=x^2+v^2+\beta _4 xv$. By the arithmetic-geometric mean inequality, $x^2+v^2\ge 2xv$, hence

$$ \Phi (x,v) \ge (2+\beta _4)xv. $$

- If $\beta _4>-2$, the right-hand side is *Φ (x,v)>0* for all *x,v>0*.
- If $\beta _4=-2$, $\Phi (x,v)=(x-v)^2\ge 0$ and vanishes along *x=v*.
- If $\beta _4<-2$, choosing *x=v* yields $\Phi (x,v)=(2+\beta _4)x^2<0$.

*□ *

##### Theorem 6

**(Isobole-level feasibility)** For any fixed *y ∈ (0,1)*, the Finney isobole

$$ c_1 + \rho c_2 + \beta _4 \sqrt{\rho c_1 c_2} = I_y $$

admits a real and nonnegative solution $(c_1,c_2)$ if and only if

$$ \beta _4^2 \;\ge \; 4\big (1 - \eta _y\big ), $$

where $\eta _y$ is a level-dependent ratio that reduces to $\eta _y = I_y/A_{y_1}$ or $\eta _y = I_y/(\rho A_{y_2})$ depending on which variable is fixed. In particular, if $|\beta _4| \ge 2$, the feasibility condition is always satisfied.

##### Proof

Set $$x=\sqrt{c_1}$$ and $$v=\sqrt{\rho c_2}$$,

- **Case A:** The isobole equation reduces to a quadratic in *v*:
  $$ v^2+\beta _4 x v+(x^2-I_y)=0 $$
  Real (hence nonnegative) solutions exist if and only if the discriminant *Δ (x)* is nonnegative:
  $$ \Delta (x)=\beta _4^2 x^2-4(x^2-I_y)=(\beta _4^2-4)x^2+4I_y \ge 0 $$
  For $$x^2=c_1\in [0,C_{y_1}],$$
  - If $$|\beta _4|\ge 2$$, then $$\Delta (x)\ge 4I_y>0$$.
  - If $|\beta _4|<2$, *Δ (x)* decreases with $x^2$ and its minimum occurs at $x^2=C_{y_1}$, yielding
    $$ 4I_y-(4-\beta _4^2)C_{y_1}\ge 0 \iff \beta _4^{2}\ge 4(1-I_y/C_{y_1}). $$
- **Case B:** Symmetrically, write the isobole equation in the quadratic in *x*
  $$ x^2+\beta _4 v x+(v^2-I_y)=0 $$
  Real (hence nonnegative) solutions exist if and only if the discriminant *Δ (v)* is nonnegative:
  $$ \Delta (v)=\beta _4^2 v^2-4(v^2-I_y)=(\beta _4^2-4)v^2+4I_y \ge 0 $$
  The same monotonicity argument on $v^2=\rho c_2\in [0,\rho C_{y_2}]$ gives
  $$ \beta _4^{2}\ge 4(1-I_y/(\rho C_{y2})). $$

Since $\rho = C_{y_1}/C_{y_2}$, these two conditions are equal to each other.*□ *

##### Corollary 4

**(Singularity lines)** Let $\Phi (c_1,c_2)$ denote the argument of the logarithm in the Finney surface model. In the positive region $(c_1,c_2>0)$ the singular set $\{\Phi (c_1,c_2)=0\}$ has the following structure:

- If $\beta _4 = -2$, the singular set reduces to the single line
  $$ c_2=\rho ^{-1}c_1, $$
  along which *Φ =0*.
- If $\beta _4 < -2$, the singular set consists of two lines
  $$ c_2=\frac{1}{\rho \,t_\pm ^{\,2}}\,c_1, \qquad t_\pm =\frac{-\beta _4\pm \sqrt{\beta _4^2-4}}{2}>0, $$
  which bound a sector where *Φ <0* (i.e. the model is undefined in that region).

##### Proof

From Theorem 6, $$\Phi (x,v) \ge (2+\beta _4)xv$$.

- If $\beta _4=-2$, then $\Phi =(x-v)^2\ge 0$, and *Φ =0* iff *x=y*, i.e., $c_2=\rho ^{-1}c_1$
- If $\beta _4<-2$, write $t=x/v=\sqrt{c_1/(\rho c_2)}$. Then
  $$ \Phi =v^2\,(t^2+\beta _4 t+1). $$
  The quadratic has two positive roots $t_\pm =\frac{-\beta _4\pm \sqrt{\beta _4^2-4}}{2}>0$, so *Φ (x,v)=0* along $t=t_\pm$, i.e., along the two lines, and *Φ <0* strictly between them.

*□ *

The feasibility analysis of the Finney model highlights that the interaction parameter $\beta _4$ plays a decisive role in determining whether the log-argument remains strictly positive. For $\beta _4 > -2$, the model is globally feasible, ensuring biologically interpretable responses across all dose combinations. At the boundary case $\beta _4=-2$, singularities appear along a single line, while for $\beta _4<-2$, entire sectors of the input space yield complex-valued responses where the model breaks down. Thus, the Finney formulation accommodates both synergistic and antagonistic interactions, but antagonism beyond the threshold $\beta _4=-2$ introduces non-feasible regions that limit its practical applicability.

## Results

The analytical feasibility conditions derived in the Methods section are illustrated in this section using representative numerical examples. The results demonstrate how different response surface models yield biologically interpretable responses, or fail to do so, depending on the model structure and parameter values. The Hill coefficient values employed in these examples are selected for illustrative purposes to highlight the feasibility properties of the models. In practical PKPD applications, Hill coefficients are typically constrained to physiologically plausible ranges, as higher values may indicate atypical binding behavior.

### Illustrative examples for Greco model

Let us consider the Greco model in Eq. 7, whose feasibility characteristics are governed by Theorem 1 (global feasibility) and Theorem 2(isobole level feasibility). For numerical illustration, the model parameters are fixed as:

$$ C_{{50}_1} = 5, \quad C_{{50}_2} = 10, \quad \gamma _1=\gamma _2=\gamma =3.5 $$

**Fig. 3:**
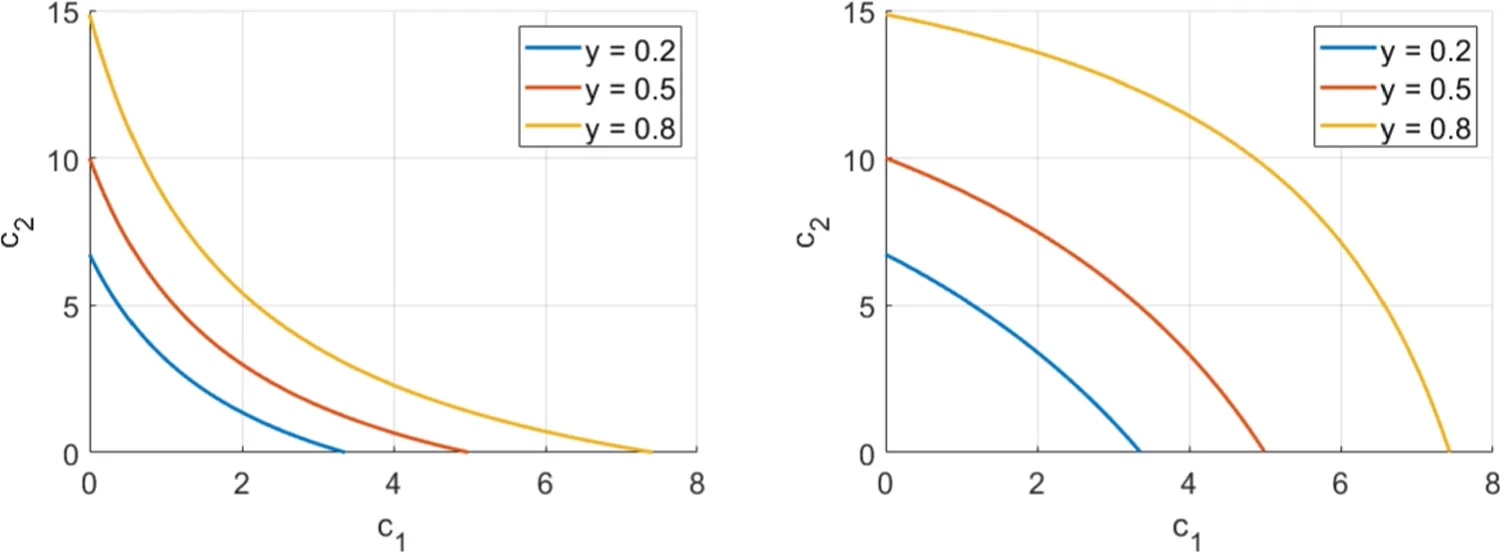
Isoboles for *β =2.5* and *β =-0.5* to produce the effect *y=0.2, y=0.5* and *y=0.8*

*Case 1: Globally feasible scenario.* For *β = 2.5*, the global feasibility condition in Theorem 1 ($\beta \ge 0$) is satisfied. In this case, the normalized index remains positive across the entire input domain, and the isoboles are well-defined. Figure 3-left illustrates three isoboles corresponding to *y = 0.2*, *y = 0.5*, and *y = 0.8*.

*Case 2: Isobole-level feasible but globally infeasible scenario.* For *β = -0.5*, the global feasibility condition in Theorem 1 ($\beta \ge 0$) is violated. However, isobole-level feasibility (Theorem 2) still holds for the three selected effect levels *y = 0.2*, *y = 0.5*, and *y = 0.8*.

Using relation Eq. 5, the corresponding input pairs $(C_{y,1}, C_{y,2})$ are (3.36, 6.72) for *y = 0.2*, (5, 10) for *y = 0.5*, and (7.42, 14.86) for *y = 0.8*. According to Theorem 2, the interaction parameter *β * ranges required for feasibility at these levels are

$$ \beta>-1.48, \beta>-1.00, \beta >-0.67, $$

respectively. As shown in Fig. 3-right, the isoboles remain well-defined for *β = -0.5*.

**Fig. 4:**
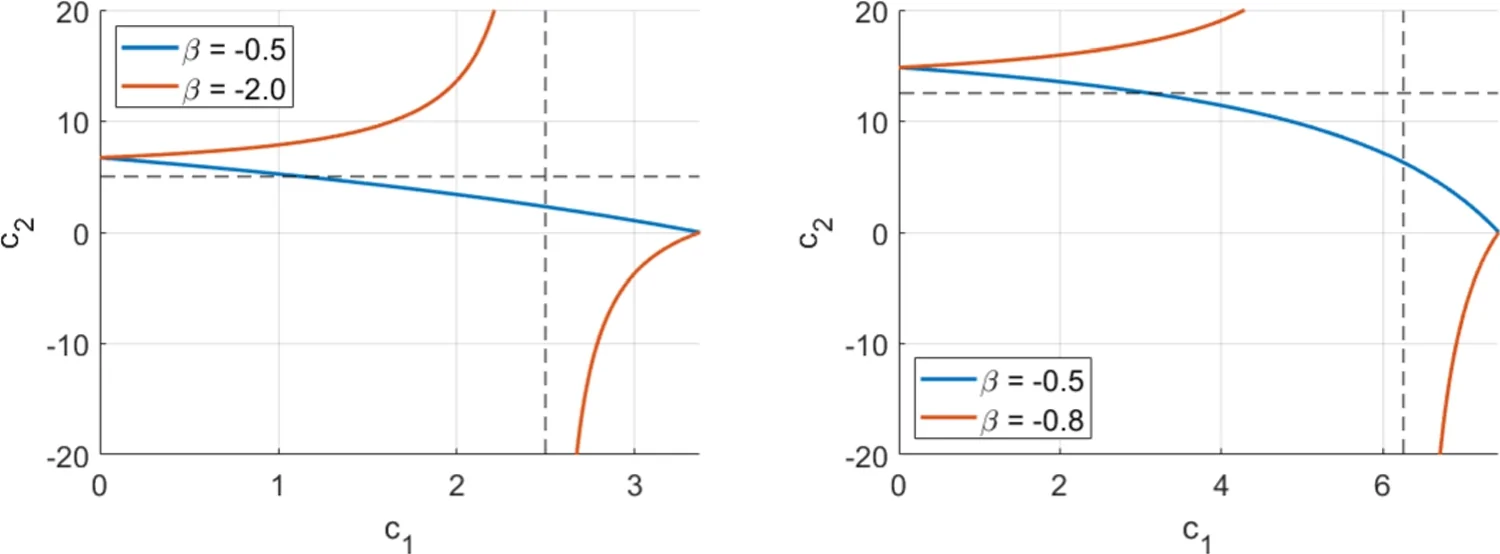
Isoboles for *β =-0.5* and *-2* to produce the effect *y=0.2* (left) and Isoboles for *β =-0.5* and *-2* to produce the effect *y=0.8* (right)

*Case 3: Both isobole-level and global infeasibility.* For *y = 0.2*, isobole-level feasibility requires *β > -1.48*. Choosing *β = -2* violates this condition, resulting in singularity lines. According to Corollary 1, the vertical and horizontal singular lines occur at:

$$ c_1 = \frac{5}{2} = 2.5, \quad c_2 = \frac{10}{2} = 5. $$

The singularity lines can be observed in Fig. 4-left, and the figure depicts the behaviour of the isoboles, highlighting the transition towards infeasibility.

For *y = 0.8*, isobole-level feasibility requires *β > -0.67*. Setting *β = -0.8* also produces singularity lines, located at:

$$ c_1 = \frac{5}{0.8} = 6.25, \quad c_2 = \frac{10}{0.8} = 12.5. $$

The singularity lines can be seen in Fig. 4-right. These singularities highlight the transition to complete infeasibility, where the model fails to produce well-defined isoboles.

### Illustrative examples for Minto model

Next, consider the Minto model in Eq. 8, whose feasibility properties are governed by Theorem 3 (global feasibility) and Theorem 4 (isobole-level feasibility). For numerical illustration, the model parameters are fixed as:

$$ C_{{50}_1} = 8, \quad C_{{50}_2} = 4, \quad \gamma _1 = \gamma _2 = \gamma = 2. $$

**Fig. 5:**
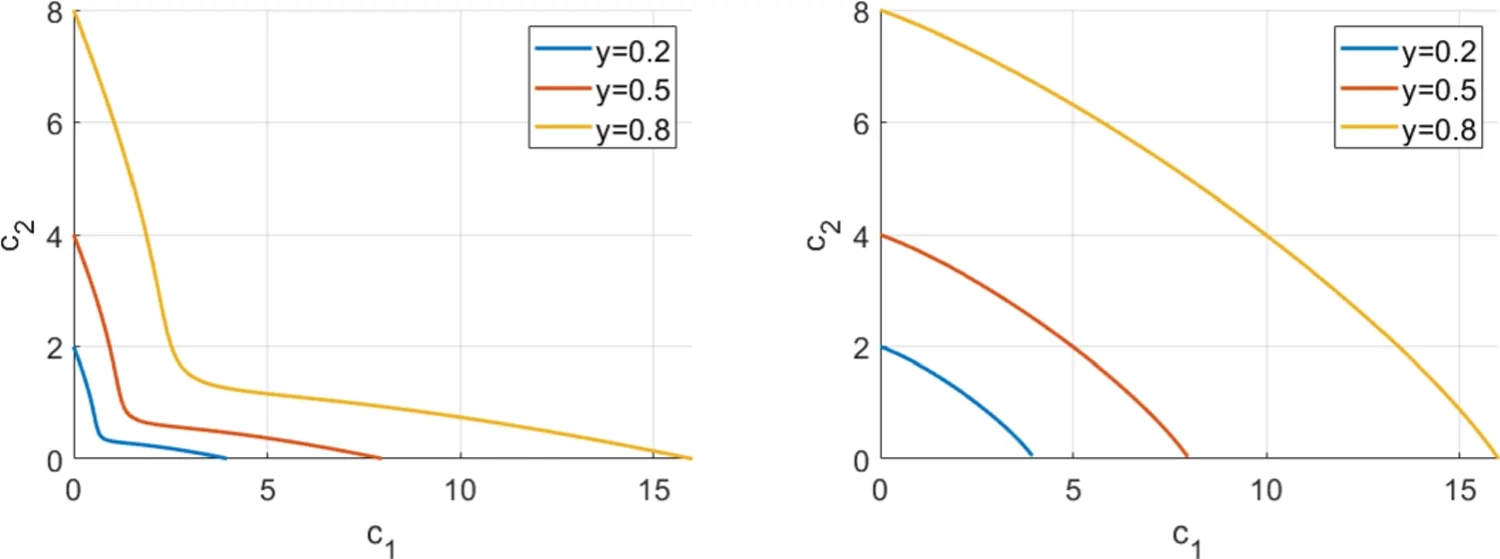
Isoboles for *β =2.5* and *β =-0.5* to produce the effect *y=0.2, y=0.5* and *y=0.8*

*Case 1: Globally feasible scenario.* For *β = 2.5* or *β = -0.5*, the global feasibility condition in Theorem 1 ($\beta \le 4$) is satisfied. Under this condition, the normalized index remains positive across the entire input domain, and the isoboles are well-defined. Figure 5 illustrates three isoboles corresponding to the effect levels *y = 0.2*, *y = 0.5*, and *y = 0.8*.

**Fig. 6:**
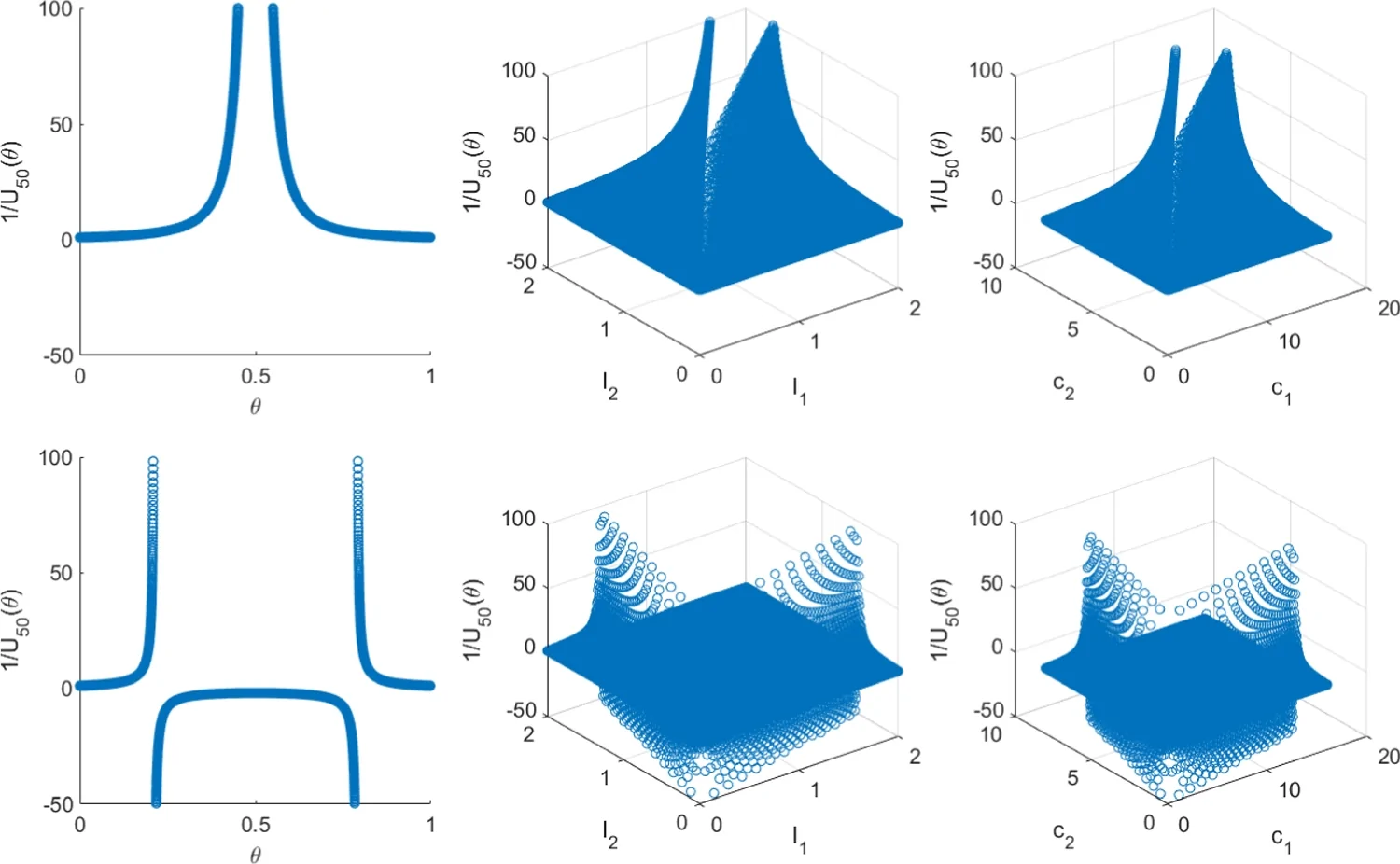
Singularity points and lines for *β =4* and *β =6*

*Case 2: Globally infeasible scenario with one singularity.* For *β = 4*, the global feasibility condition in Theorem 3 is no longer satisfied. In this case, there exists a single singular point at *θ = 0.5*. According to Corollary 3, the singularity line in the $(I_1, I_2)$ plane is:

$$ I_1 = I_2, $$

and according to Corollary 4, the corresponding singularity line in the $(c_1, c_2)$ plane is:

$$ c_2 = \frac{4}{8} c_1 = 0.5\,c_1. $$

These results are illustrated in Fig. 6-top.

*Case 3: Globally infeasible scenario with two singularities.* For *β = 6*, the global feasibility condition in Theorem 3 is violated. This leads to two singular points, located at *θ = 0.21* and *θ = 0.78*. According to Corollary 3, the corresponding singularity lines in the $(I_a, I_b)$ plane are:

$$ I_1 = 3.73\,I_2, \quad I_1 = 0.26\,I_2, $$

and according to Corollary 4, the singularity lines in the $(c_1, c_2)$ plane are:

$$ c_2 = 1.86\,c_1, \quad c_2 = 0.13\,c_1. $$

These results are illustrated in Fig. 6-bottom.

### Illustrative examples for Finney model

We finally consider the Finney model in Eq. 11, whose feasibility properties are characterized by Theorem 5 (global feasibility) and Theorem 6 (isobole-level feasibility). For numerical illustration, the model parameters are fixed as:

$$ \beta _0 = 0, \quad \beta _1 = 2, \quad \rho = 2. $$

**Fig. 7:**
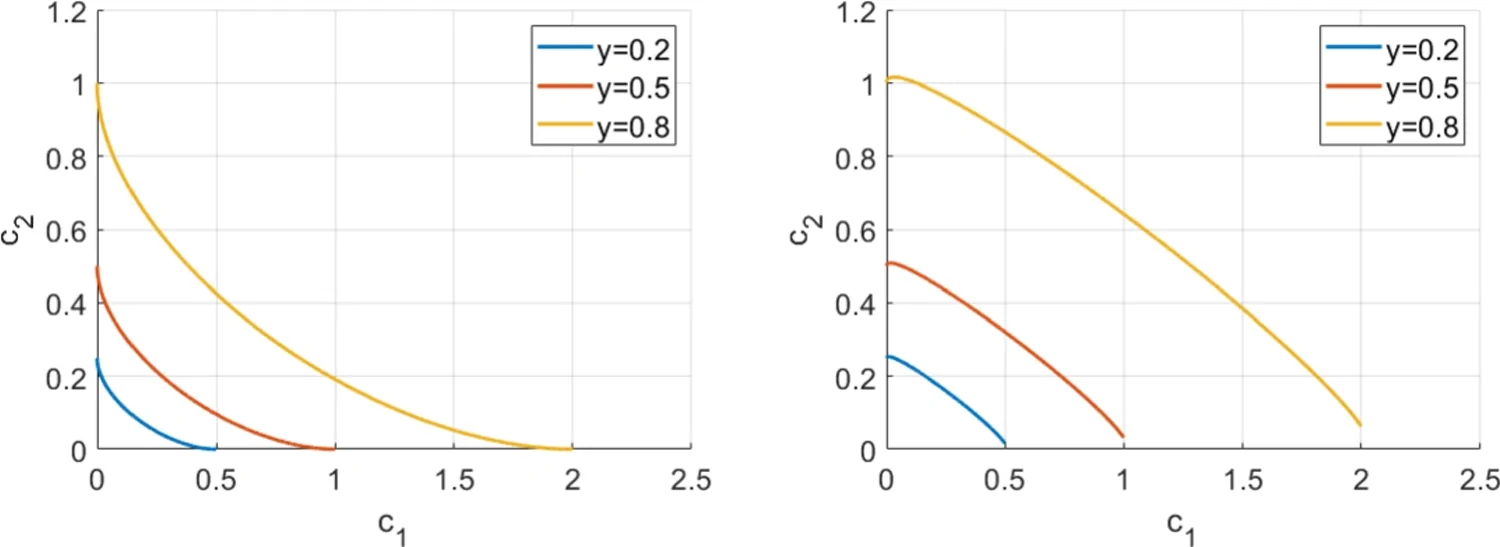
Isoboles for $\beta _4=1$ and $\beta _4=-0.25$ to produce the effect *y=0.2, y=0.5* and *y=0.8*

*Case 1: Globally feasible scenario.* For values of $\beta _4 = 1$ or $\beta _4 = -0.25$, the global feasibility condition in Theorem 5 ($\beta _4 \ge -2$) is satisfied. Under this condition, the normalized index remains positive across the entire input domain, and the isoboles are well-defined. Figure 7 illustrates three isoboles corresponding to the effect levels *y = 0.2*, *y = 0.5*, and *y = 0.8*.

*Case 2: Globally infeasible scenario with one singularity.* When $\beta _4 = -2$, the global feasibility condition in Theorem 5 ($\beta _4 \ge -2$) is violated at the boundary. According to Corollary 4, this results in a single singularity line in the $(c_1,c_2)$ plane:

$$ c_2 = 0.5\, c_1. $$

**Fig. 8:**
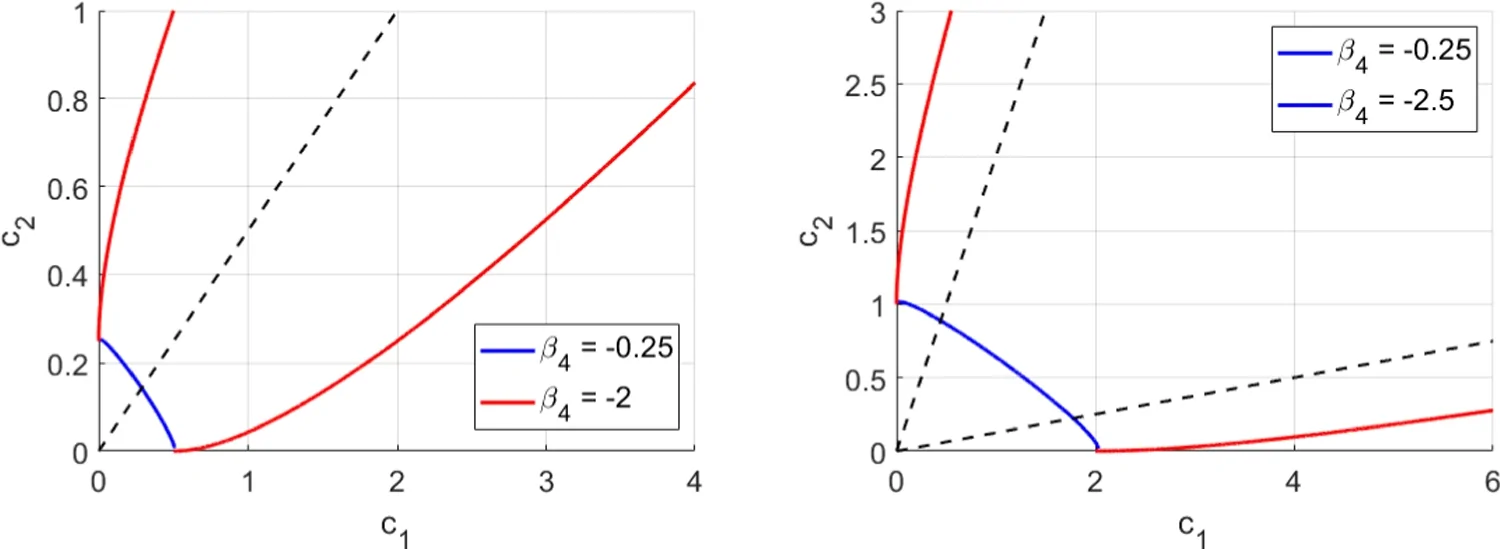
Isoboles for *β =-0.25*, and *β = -2* to produce the effect *y=0.2* (left) and Isoboles for *β =-0.25* and *β =-2.5* to produce the effect *y=0.8* (right)

This behaviour is illustrated in Fig. 8-left.

*Case 3: Globally infeasible scenario with two singularities.* For $\beta _4 = -2.5$, the global feasibility condition in Theorem 5 is violated. In this case, two singularity lines emerge in the $(c_1,c_2)$ plane based on Corollary 4. The roots are $t_\pm = \frac{-\beta _4 \pm \sqrt{\beta _4^2-4}}{2}$, yielding $t_- = 0.5$ and $t_+ = 2$. Using $c_2 = \frac{1}{\rho \, t_\pm ^{2}}\,c_1$ with *ρ =2*, the singularity lines are

$$ c_2 = 2\,c_1 \quad \text {and}\quad c_2 = 0.125\,c_1. $$

These results are shown in Fig. 8-right.

## Discussion

This work presented a systematic feasibility analysis framework for response surface interaction models, with the aim of clarifying under which structural and parametric conditions these models yield biologically interpretable responses. By explicitly distinguishing between isobole-level feasibility and global feasibility, the proposed framework provides a structured way to assess whether model predictions remain well-defined locally at fixed effect levels or across the entire input domain.

The model-specific analyses demonstrate that feasibility is not an inherent property of response surface formulations, but rather a consequence of the interplay between model structure and interaction parameters. For the Greco model, the results indicate that global feasibility is guaranteed only under non-antagonistic interaction regimes. While the model can formally represent both synergistic and antagonistic interactions, antagonism restricts feasibility to limited regions of the dose–effect surface, with singularity lines emerging within the positive quadrant. This highlights that practical use of the Greco formulation in pharmacological or clinical settings requires careful parameter selection to ensure biological interpretability over the relevant dose range.

A similar sensitivity to interaction strength is observed for the Minto model. Moderate interaction parameters preserve global feasibility, whereas stronger interactions induce singularities in both normalized and input domains. These singular structures reveal intrinsic limitations of the formulation and identify parameter regimes where biologically interpretable responses can no longer be guaranteed. Consequently, reliable application of the Minto model necessitates avoiding interaction regimes that give rise to such singular behavior. For the Finney model, feasibility is governed by the positivity of the logarithmic argument. While the model remains globally feasible for moderate interaction parameters, stronger antagonism introduces singular boundaries and regions with complex-valued responses. This observation underscores that, although the Finney formulation is flexible enough to capture both synergistic and antagonistic interactions, excessive antagonism fundamentally limits its domain of biological validity. The numerical illustrations support the analytical findings and demonstrate how feasibility boundaries manifest in practice. Importantly, these examples emphasize that RSMs may yield well-defined and biologically interpretable responses only within specific parameter regimes, even when the underlying mathematical expressions appear formally valid. This reinforces the need for explicit feasibility analysis when applying such models to PKPD and related applications.

The choice of Hill coefficient values in the numerical examples also merits discussion. In this study, Hill coefficients were selected for illustrative purposes to expose feasibility boundaries and to highlight the effect of increasing nonlinearity on response surface behavior. In practical PKPD applications, however, Hill coefficients are typically constrained to physiologically plausible ranges, as larger values may indicate atypical or problematic binding behavior. This distinction clarifies that the numerical examples are intended to support methodological insights rather than to represent specific pharmacological binding mechanisms.

Taken together, these results demonstrate that feasibility analysis provides a valuable complement to traditional response surface modeling approaches. By explicitly identifying parameter regimes that preserve biological interpretability and excluding regions associated with singular or complex-valued responses, the proposed framework offers practical guidance for model selection, parameter tuning, and interpretation.

Finally, while this study focused on two-agent interaction models, an important direction for future research is the extension of the proposed feasibility framework to multi-agent systems involving three or more inputs. Such extensions would require reformulating interaction terms and feasibility conditions in higher-dimensional domains, posing both mathematical challenges and practical opportunities for combination therapy and other complex multi-agent applications.

## Data Availability

No datasets were generated or analysed during the current study.
